# Supplementary figures and images for: Cross-platform mechanical characterization of lung tissue
Source: PLoS One. 2018 Oct 17;13(10):e0204765. doi: 10.1371/journal.pone.0204765 (PMC6192579; doi:10.1371/journal.pone.0204765)

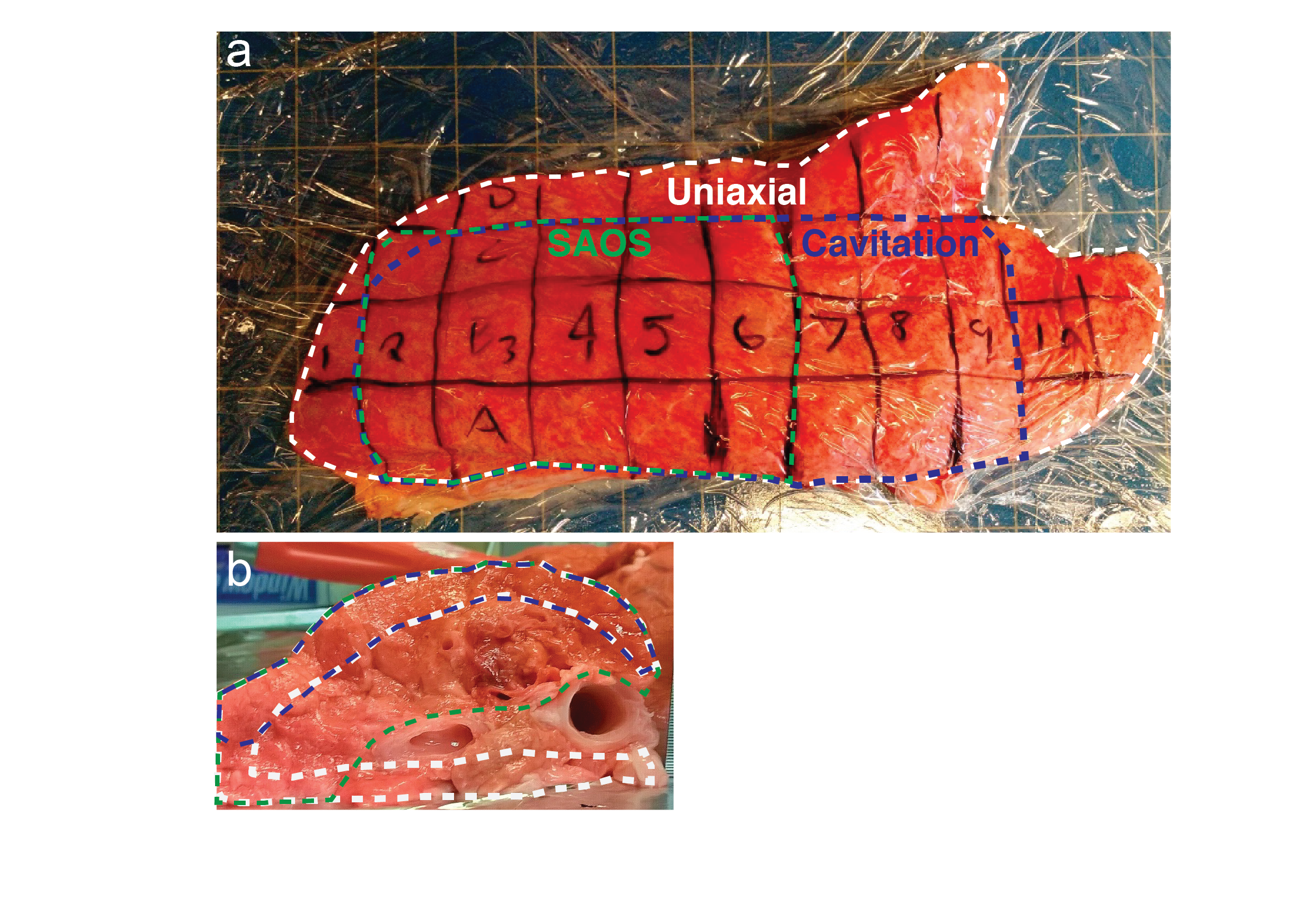

Supplement: S1 Fig — (a) Shown is an example of the grid system used for marking the lung tissues prior to testing and/or excision. The lungs were wrapped in plastic wrap and a grid consisting of 1in x 1in squares was drawn onto the wrap in order to help identify lung samples. (b) This image shows the inner structure of the lung (near column 4 in (a)), which is extremely heterogeneous and cartilage-rich that had to be avoided during tissue excision for SAOS, micro-indentation, and uniaxial tension. The colored, dashed lines denote the tissue areas used for each technique. Micro-indentation can be taken from anywhere within the lung, but there are restrictions on where samples can be taken for other tests such as cavitation (blue), uniaxial testing (white) and SAOS (green). Samples were excised as outlined in the dashed lines in (a) and (b) for each method, avoiding the cartilaginous bronchioles. (TIF) [file pone.0204765.s001.tif]

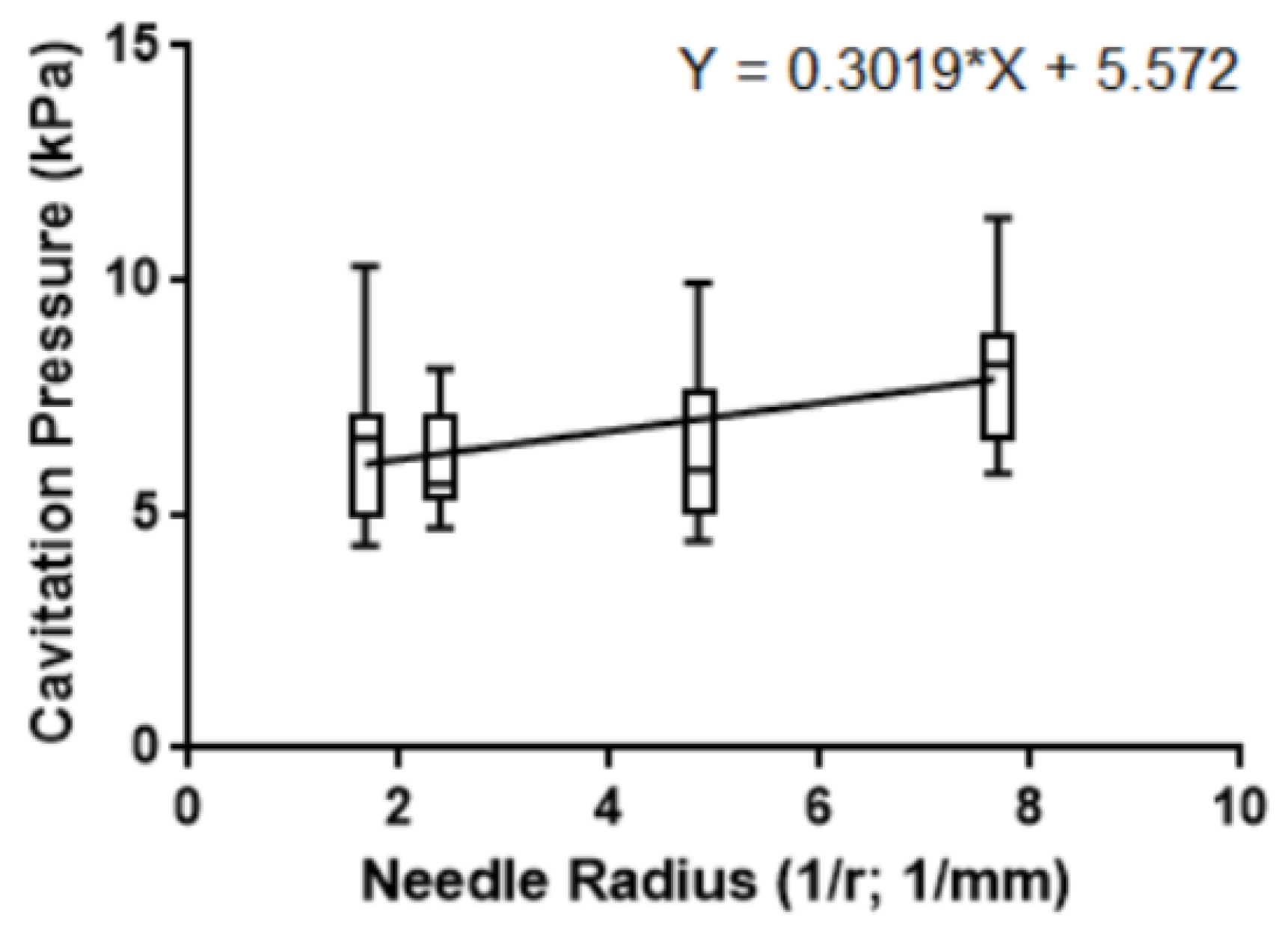

Supplement: S2 Fig — The Young’s modulus of the sample can be found by fitting a line to the data and finding the intercept (5.6±0.5 kPa). Error bars represent the standard deviation. The intercept can be used to determine the elasticity of the material. (TIF) [file pone.0204765.s002.tif]

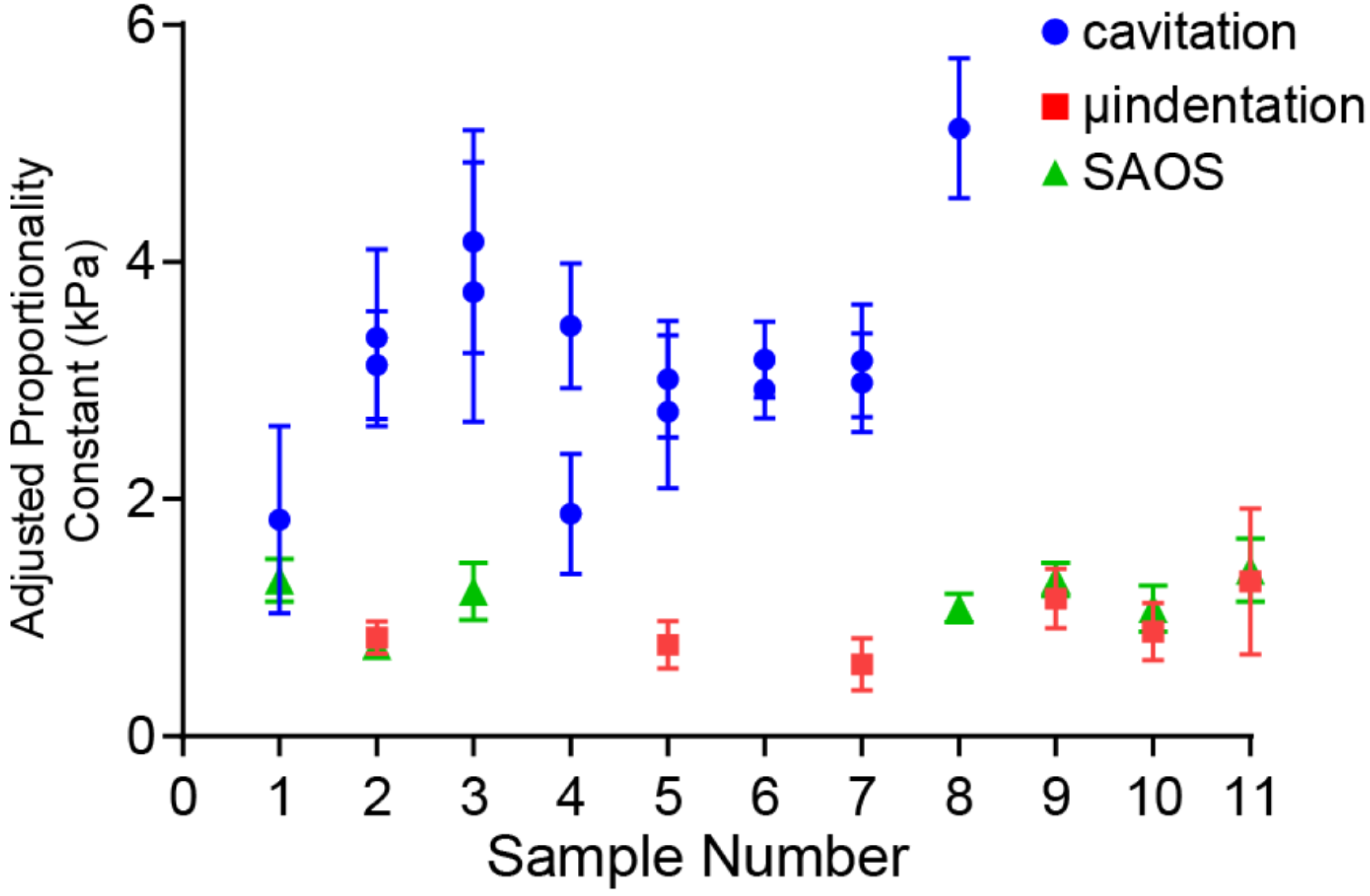

Supplement: S5 Fig — The Young’s moduli were adjusted based on the power law to compensate for the differences in the frequency of the tests. (TIF) [file pone.0204765.s005.tif]
